# Highlights from the 2016 WIN Symposium, 27–29 June 2016, Paris: personalised therapy beyond next-generation sequencing

**DOI:** 10.3332/ecancer.2016.669

**Published:** 2016-08-24

**Authors:** Richard Schilsky, Will Davies

**Affiliations:** 1 American Society of Clinical Oncology, Alexandria, Virginia 22314, USA; 2 Cancer Intelligence, 154 Cheltenham Road, Bristol, UK BS6 5RL

**Keywords:** personalised medicine, genomics, biomarkers, targeted therapy, sequencing

## Abstract

The Worldwide Innovative Networking (WIN) consortium is an alliance of academic institutions, pharmaceutical partners, representatives from technology companies and charitable/health payer organisations from across the globe. For the last six years, the consortium’s aims have been to foster communication and collaboration between members, encourage dialogue in an open forum, and deliver clinical trial results that improve the care and outcomes of patients with cancer using the latest advances in genomic-based medicine.

The annual WIN Symposium, held over two days, is a chance for its members to come together and discuss ongoing research, recent announcements, and introduce new developments in personalised medicine. This year’s conference, held in Paris, France 27–29 June, consisted of six dedicated sessions, including two debates, and posters from members and participating organisations, all focusing on the latest therapeutic advances and updates in genomic analysis.

Special highlights from this year included discussion of the MINDACT clinical trial, which uses a gene expression test to identify patients with breast cancer who can safely forego adjuvant chemotherapy, and the reflections on the SHIVA trial. Of particular interest to many speakers was the utilisation of liquid biopsy samples to produce near real time snapshots of tumour mutational profiles and vulnerability.

Following a welcome from WIN chairman Dr John Mendelsohn (The University of Texas MD Anderson Cancer Centre, Houston, TX, USA), the first session focused on considerations of patient perspectives by Dr Francesco de Lorenzo (European Cancer Patient Coalition, Brussels, Belgium) who called for participation, advocacy, and awareness from healthcare providers and Matt Ellefson (SURVIVEiT, USA). Matt, who is living with stage four lung cancer, founded SURVIVEiT following his own diagnosis so as to better connect patients with support networks and available practitioners, and to encourage dialogue between patients and doctors over the course of their treatment. With a SURVIVEiT mobile application now launching, Dr Lorenzo and Matt Ellefson both made the case that personalised care is not encapsulated in the choice of treatment, but is reflected at every stage of care.

The second session, ‘New Tools for Early Diagnosis, Selecting Therapies, and Monitoring’ opened with Prof Andrea Califano (Columbia University Medical Centre, New York, USA), speaking in his capacity as founder of Darwin Health. He began by addressing the successes of matching therapy to known mutational drivers of cancer, and he also acknowledged the limitation in narrowly treating a single driver mutation with single agent therapies. Discussing the process through which genomic characterisation of patients helps tailor treatments, he presented results on how drug sensitivity can be identified, via RNA sampling and protein activity, where no mutation is apparent using the VIPER algorithm [1]. He also gave updates on the identification and understanding of so-called DNA master regulators he addressed in the previous year’s conference. He identifies these as highly conserved and regulated areas of genes which may well be the wellspring from which so many other cancers develop, or may be prevented from developing if their cascade is stemmed at the regulatory node.

The next speaker was Dr Elaine Mardis (Washington University, St Louis, USA), who elaborated further on the new tools described by Dr Califano, moving to the selection of therapies. She presented steps at which next generation sequencing could support treatment decisions, providing lowered costs, and the potential for increased patient benefit. Throughout her talk, barriers that may hinder access and interpretation of these results were brought forth. She gave examples of differing models of insurance and reimbursement for testing between countries, the uncertainty in establishing whether there is a definite benefit in offering sequencing of a tumour to all patients, and the impact of wealth disparity on diagnosis. Another such stumbling block addressed was, how with such tremendous amounts of data being generated per patient, can one clinician or even one lab filter out the noise and find something of use? Her answer was that they cannot; but that definitely an open crowdsourcing of genomic analysis and patient outcomes could lead to valuable new insights.

Rounding out this session came a comprehensive review of advances in monitoring and surveillance of patient responses, with liquid biopsy taking the centre stage. Dr Nitzan Rosenfeld (Cancer Research UK, UK) spoke on circulating tumour DNA as an indicator of changing tumoural DNA expression with the genotype seemingly as fluid as the samples themselves. Dr Ajay Goel (Baylor University Medical Centre, USA) followed with a related presentation. They shed light on the fact that using micro RNAs recovered from patient serum samples can be a good diagnostic tool, particularly in gastrointestinal malignancies. These samples can be used for monitoring of known lesions as Dr Rosenfeld suggested and as Dr Goel reported of their potential utility in early diagnosis. In both cases, their guidance to personalised medicine in treating the genetically distinct tumour of each patient, at the time of assaying, could prove invaluable even if expensive.

The theme of ongoing surveillance was continued by Dr Raghu Kalluri (University of Texas MD Anderson Cancer Centre, USA) who spoke on exosomes; the trillions of circulating vesicles that as per his report may play a role in the spread of tumour DNA and metastasis. Working with these ‘garbage bags’ of the cell, the encapsulated DNA could be a reliable summary from numerous tumour sites aiding in informing design and repurposing of personalised therapies.

Last to speak in this plenary session was Dr Alberto Bardelli (Candiolo Cancer Institute, Italy), who presented a considered evolutionary outlook on tumour tracking and development of resistance. In his work with liquid biopsies and metastatic colorectal cancer, Dr Bardelli described genomic targeting of known oncogenes including mutant HER-2, TRK, and ALK genes, along with identifying a shifting tide of susceptibility and resistance in patients. In his presentation, he compared how this acknowledgement of clonal evolution is coming late in the day, considering the history of cellular mutations that have led to the very biodiversity we ourselves are part of. Speaking later in an interview, he said ‘I was pretty shocked by how poor we had previously made parallels between evolution of cells that had been living on this Earth for billions of years and tumour cells. We shouldn’t be surprised by that, but we are in a way, and we’ve rediscovered this principle law of biology…’ His schema of biopsy collection, based on biweekly blood draws, is also reported as being more convenient for patients, and Dr Bardelli said he looked forward to a future in which blood samples, or perhaps even urine are enough to start a patients’ cancer care journey.

The third session of the conference shifted focus from technology in practice to review recent clinical trials, reporting on results which were thought to substantially improve outcomes. Dr William Sellers (Novartis Oncology, USA) began with a reflection on the combinatorial success of ABL001, first reported at ASH 2014 [2].

The focused path to reaching ABL001 combinations stood in contrast to a broader perspective from Dr Susan Galbraith (AstraZeneca, UK). Looking at cancers which can be effectively ‘cured’ currently, she described the impact of combination therapies in managing the development of resistance, and how best to manage tolerable doses of multiple drugs simultaneously. With intermittent scheduling to keep cancer cell recovery off balance, Dr Galbraith summarised how ‘hit and run’ therapy is delivering partial and complete responses rates in an overall shorter time frame when compared to long-term survival with the risk of resistance and relapse reduced compared to using static monotherapy. With emerging immunotherapeutic agents, including cell checkpoint inhibitors, now entering doublet and triplet trials, and PARP inhibitors offering means to completely box in the mechanisms of tumour evolution and spread, Dr Galbraith’s work seems to be putting the combination credo of the WIN consortium into active practice.

On the subject of immunotherapy, the last speaker in this plenary session, Dr Daniel de Carvalho (Princess Margaret Cancer Centre, Canada), gave his experience and perspective in how epigenetic therapies can contribute to diagnosis and design of care. When considering the potential of viral mimicry in directing and managing patient immune response, the question was raised if such methods constituted a ‘chronic infection’ paradigm of treatment. Given the short half-life and low penetration of viral agents tested so far, he described monthly treatments as manageable, non-exhausting, and nonstimulating of any autoimmune side effects in mouse models. He presented an example of this pairing in action as the METADUR trial, a phase II basket study combining the checkpoint inhibitor durvalumab with hypomethylating azacitidine in solid tumours, with patient enrolment opening soon.

The final session of the conference’s first day may yet go down in WIN history as one of the most contentious, as Dr Christophe Le Tourneau (Institut Curie, France) and Dr Razelle Kurzrock (University of California San Diego, USA) took to the stage to debate whether the current design of precision medicine trials is the right one. The case up for discussion was the SHIVA trial which, unexpectedly, determined precision medicine had no effect [3].

The design of the SHIVA trial was such that, between the differing arms, patients with an identified mutation for which a specific therapy is available would receive either targeted therapy based on molecular profiling, such as imatinib, trastuzumab, or letrozole, or receive conventional therapy at their physician’s discretion. It is worth noting at this point that patients were able to cross over between arms. In assessing the progression-free survival (PFS) of patients over 12 months, both arms were found to offer similar outcomes, meaning molecular profiling and targeted treatment assigned via a pre-specified algorithm offered no improvement over physician choice of treatment.

Dr Le Tourneau opened with a defense of SHIVA, which he described as ultimately an algorithm testing trial. Deferring from the question of whether the design of the trial was right, he posed that it addressed a question which would come to the mind of many physicians and patients ‘Is it worth $5K to get a molecular profile? Will that improve my outcome?’ He also highlighted the trial most directly interrogated the algorithm itself, for which the aspects of available technology, quality control, validation, and interpretation must all be considered. Amongst this, he noted that the cross-over data favours switching from physician’s choice to molecularly targeted therapy. In summary, Dr Le Tourneau asserted that the SHIVA trial is a relevant design strategy and that using PFS ratio as a primary end point would be a more direct comparison of patient arms. Most of all, refinement of treatment algorithms is where he saw the most work to be done.

However, even before the counter-argument could be presented, a concern raised by an audience member was that the majority of patients sorted through the algorithm received single agent everolimus or hormone treatment, and this is something that does not in itself reflect the combinatorial capabilities of most precision medicines, nor it is something that physicians would select for advanced cancer. Further concerns were raised over questionable matches made by algorithmic selection, the evolution of technology within the time constraints of the trial, and whether randomised trials are still the gold standard of assessment. Most critically to the integrity of the SHIVA defense is that, between algorithmic assignment of treatment and physician’s choice, there is more than one randomised element at stake—both arms ought to receive physician’s choice or personalised medicine to truly assess the algorithm and its matches. As Dr Kurzrock later commented, ‘Unmatched targeted therapy is one of the worst things you can do to a patient’.

In her critique of the trial, Dr Kurzrock asked few questions of Dr LeTourneau and the SHIVA design, but the answers stimulated greater discussion. When asked how many patients are needed for a retrospective trial such as this, when addressing every mutation from each tumour of all patients, debate among the audience varied from n-of-1 for fully personalised treatment to discussing the value of population wide data libraries. As for the readiness of widespread adoption of personalised treatment, one speaker commented ‘We are ready to go right into precision medicine for all, with no proof it works and no mechanism to record the outcomes’. Speaking as chair of the debate, I commented ‘SHIVA… is not an indictment of the field of precision medicine, only an illustration of how complicated it is, and we need to continue to do research.’

With discussion still rippling through the crowd as the formal debate finished, the first day of programmed talks wound to a close. From the conversations that lingered among attendees that evening we reignited the following morning; the cases for and against SHIVA were far from laid to rest.

The second day of the conference and the fourth plenary session sought to review relevant models and preclinical data. The session began with the keynote lecture from Dr Leroy Hood (Institute for Systems Biology, USA), whose attitudes to bringing ‘systems biology’ to the forefront of patient care illustrate the widest reaches of innovation discussed at WIN. From introducing his conceptual P4 medicine strategy—predictive, preventive, personalised, and participatory care—it is clear that the clinical ablation of cancerous cells is a small part of his idea of personalised medicine.

A key aspect of his vision of care is utilising the full breadth of patient data, or ‘data clouds’ as he identified them, to begin with identifying specific protein or protein network aberrations to diagnose and track patient disease states. To achieve such holistic care, he outlined some novel technologies that he hopes can maximise data interrogation, from the genomic sequencing beyond ‘Next Generation’ abilities, to targeted mass spectrometry and highly multiplexed assaying of single cells, to peptide protein capture systems, which he estimated will ‘replace monoclonal antibodies’. With regards to liquid biopsies and blood based diagnosis, he described successes with a pulmonary nodule assay, which he reported was making significant savings and already helping patients ranging from preterm births to veterans with posttraumatic stress disorder (PTSD).

In closing, he again addressed his hope for Scientific Wellness to become the default state for health care, in so much as it is cheaper, easier, and more effective to assure ongoing patient health than expectant waiting for disease to manifest.

An issue widely accepted within genomic screening is how best to interrogate and sort the wealth of data available in a patient’s sequencing results. Considering the established significance of EGFR and HER-2 mutations in lung and breast cancer, Dr Livio Trusolino (Candiolo Cancer Institute, Italy) reported on oncogenic drivers, such as IGF2 identified in colorectal cancer patients while working on known biomarkers across a wide constellation of cancers. Dr Trusolino described how the presence of known indicators of tumourigenesis from other sites in colorectal cancer samples offers a well-informed starting point, and the importance of individualised sensitivity testing to reflect the heterogeneity of cancer from patient to patient. In closing, he introduced results from phase II of the HERACLES trial in which HER-2+, KRAS wt mCRC’s response to combined trastuzumab and lapatinib were shown.

Next, Dr Robert Vries (Foundation Hubrecht Organoid Technology, the Netherlands) spoke on his work with organoids as models. His lab is well placed to research organoids, given they were pioneered there by Prof Hans Clevers who spoke about them at WIN 2014 [4]. With organoids’ clonal reliability, genetic stability, and long-lasting viability, Dr Vries spoke on how the symposium focus of personalised medicine through genomic screening and drug targeting is streamlined in research and application using organoids as a possible replacement for animal models.

Dr Jean-François Martini (Pfizer Inc., USA) added another voice to the choir supporting the use of liquid biopsies, using circulating cell-free DNA to determine patient genotypes over the course of the PALOMA 3 trial [5], which assessed fulvestrant with either palbociclib or a placebo, and a separate first-in-human trial of lorlatinib for ALK+ non-small cell lung cancer (NSCLC) [6].

The morning’s session rounded out with a presentation of the most recent results from the MINDACT clinical trial from Dr Suzette Delaloge (Gustave Roussy, France). The MINDACT trial has attracted attention since its inception [7], and the interest has intensified since the first publication of results at this years’ AACR conference; there exist patient subtypes for whom a 70 gene signature is a better predictor of their need for chemotherapy than Adjuvant! Online assessment. The patient benefits of being spared chemotherapy are apparent, and Dr Delaloge lends equal consideration to the socioeconomic burden when lifted in terms of time and cost for healthcare providers and recovery. Of particular note is the 92% threshold for ten year OS, and that even in discordant (clinical high risk, genomic low risk) patients, a five year distant metastasis-free survival (DMFS) rate over 94% was observed. Overall, Dr Delaloge described an overwhelming success in reducing the prescription of chemotherapy while maintaining patient survival, and further results will be reported this December in the San Antonio Breast Cancer Symposium. Similar sentiments were echoed later in the afternoon by Dr Funda Meric-Bernstam (University of Texas MD Anderson Cancer Centre, USA), whose research into genotyping breast cancer patients and patient derived xenografts is being guided by algorithmic sorting of sensitivities to novel treatments.

The next session was given over to a panel of some of the leading voices in personalised cancer care and from research institutions around the world. Among them, Dr Tomasz Tursz (Fondation ARC, France) brought his focus especially towards pharmaceutical companies that might restrict access to drug data from phase I trials that do not proceed. Highlighting the ‘unknown unknowns’ of data that might contribute to novel combinations, and the value of negative data, he called on clinicians present to request and participate in open sharing of data between companies, publishers, and patient advocates.

The final session for the conference sought to bring together the genomic advances discussed over the weekend with the patient clinical advances and patient outcomes that opened it. The final keynote of the conference came from Prof Bruce Johnson (Dana-Farber Cancer Institute, USA), who presented the impact of next-generation sequencing on lung cancer treatment. Having worked on lung cancer from the first use of epidermal growth factor receptor (EGFR) in patient genotyping, Prof Johnson offered insights into new targets, including MET and BRAF mutations, and how new sequencing technologies are matching patients to novel treatments with boosted survival rates from the French Cooperative Thoracic Group.

Dr Rodrigo Dienstmann (Vall d’Hebron, Spain and Sage Bionetworks, USA) spoke next on molecular signatures in colorectal cancer, and how these subtypes were translating to therapeutic opportunities. Opening with the admission that until recently colorectal cancer had few actionable biomarkers, of which only RAS mutations seem to have clinical utility, Dr Dienstmann set out his ambition for the near and far future of multi-marker and multi-omics testing, from which combined or adaptive drug therapies can be derived. Considering the <5% response from matched single agents, the wealth of co-occurring mutations and shifting heterogeneity within tumour cells as resistance develops, Dr Dienstmann spoke of the need for deeper understanding of cancer lesions through transcriptomic classification. This which would make for a more fully realised and personalised map of the active cancer pathways and immune responses.

The finale of the conference was a second debate, with arguments from Dr Yves Lussier (The University of Arizona Cancer Centre, USA) and Dr Gordon Mills (University of Texas MD Anderson Cancer Centre, USA), as to the pros and cons respectively on the controversial question, i.e., if Big Data is ready to improve patient outcomes or was it to quote the session title, ‘a new generation of garbage in/garbage out’.

In his defence of Big Data, Dr Lussier highlighted the necessity of translating the raw data generated by multi-omics into valuable information, and further translate it into articulate and actionable knowledge. With due consideration of the problems facing current data management—algorithmic behaviours, sifting useful data and patterns out of the noise, and data storage/infrastructure to begin with—Dr Lussier made a compelling case for the transformative potential of fully mined clinical data to inform case-by-case personalised medicine.

Dr Mills did nothing to dissuade this conclusion, but challenged about conflating potential with results, opening with the simple statement that ‘We aren’t ready yet’. His description of current efforts as messy data in a magic box from which results are conjured drew a nod from some audience members. The difference between potential and results was contrasted in his example of The Cancer Genome Atlas [8], an international multi-million dollar collaboration to sequence and store genomic data from tumour samples, which had ultimately led to numerous withdrawals from participating clinicians because of the lack of usable outcomes.

This was not to be taken as a pillory of collaborative genomic efforts, but as an appeal for investment from industrial informatics across international boundaries, and that when given time for technology we can trust it to mature. Referring to the issues which hampered the TCGA, i.e., Dr Mills titular argument—that we aren’t ready yet—was aimed at only knowing that if Big Data is to be widely adopted and incorporated into patient care. If yes, then it should be done right away as a misplaced first step could relegate genomics to languish without the level of funding it requires. As the central slide in his presentation, he stated, ‘You don’t want to look back and know you could’ve done better’.

## Conclusion

The debate which followed the presentations edged towards the existential—if we are all here, it must be worth someone’s time and money—and the reconciliation between doctors Dr Mills and Dr Lussier, that whether ready or not, Big Data is happening. With that tentative look towards the future of personalised medicine, the conference drew to a close. Dr Mendelsohn offered his thanks to the speakers, participants, and audience of this year’s conference, and extended his invitation to return next year.
